# Constrictive pericarditis in the setting of repeated chest trauma in a mixed martial arts fighter

**DOI:** 10.1186/s12872-021-02378-8

**Published:** 2021-11-22

**Authors:** Meganne N. Ferrel, Sentia Iriana, I. Raymond Thomason, Christy L. Ma, Katsiaryna Tsarova, Brent D. Wilson, Stephen H. McKellar, John J. Ryan

**Affiliations:** 1 University of Utah School of Medicine, Salt Lake City, UT 84132 USA; 2 Division of Gastroenterology, Department of Medicine, University of Utah, Salt Lake City, UT 84132 USA; 3 Division of Cardiovascular Medicine, Department of Medicine, University of Utah Health, 30 North 1900 East, Room 4A100, Salt Lake City, UT 84132 USA; 4 Division of Cardiothoracic Surgery, Department of Surgery, University of Utah, Salt Lake City, UT 84132 USA

**Keywords:** Case report, Constrictive, Diastolic heart failure, Hemodynamics, Imaging

## Abstract

**Background:**

Constrictive pericarditis (CP) is characterized by scarring and loss of elasticity of the pericardium. This case demonstrates that mixed martial arts (MMA) is a previously unrecognized risk factor for CP, diagnosis of which is supported by cardiac imaging, right and left heart catheterization, and histological findings of dense fibrous tissue without chronic inflammation.

**Case presentation:**

A 47-year-old Caucasian male former mixed martial arts (MMA) fighter from the Western United States presented to liver clinic for elevated liver injury tests (LIT) and a 35-pound weight loss with associated diarrhea, lower extremity edema, dyspnea on exertion, and worsening fatigue over a period of 6 months. Past medical history includes concussion, right bundle branch block, migraine headache, hypertension, chronic pain related to musculoskeletal injuries and fractures secondary to MMA competition. Involvement in MMA was extensive with an 8-year history of professional MMA competition and 13-year history of MMA fighting with recurrent trauma to the chest wall. The patient also reported a 20-year history of performance enhancing drugs including testosterone. Physical exam was notable for elevated jugular venous pressure, hepatomegaly, and trace peripheral edema. An extensive workup was performed including laboratory studies, abdominal computerized tomography, liver biopsy, echocardiogram, and cardiac magnetic resonance imaging. Finally, right and left heart catheterization—the gold standard—confirmed discordance of the right ventricle-left ventricle, consistent with constrictive physiology. Pericardiectomy was performed with histologic evidence of chronic pericarditis. The patient’s hospital course was uncomplicated and he returned to NYHA functional class I.

**Conclusions:**

CP can be a sequela of recurrent pericarditis or hemorrhagic effusions and may have a delayed presentation. In cases of recurrent trauma, CP may be managed with pericardiectomy with apparent good outcome. Further studies are warranted to analyze the occurrence of CP in MMA so as to better define the risk in such adults.

**Supplementary Information:**

The online version contains supplementary material available at 10.1186/s12872-021-02378-8.

## Background

This case demonstrates that mixed martial arts (MMA) with recurrent chest wall trauma can be a previously unrecognized risk factor for constrictive pericarditis (CP). CP is characterized by scarring and loss of elasticity of the pericardium. The most common etiologies of CP in Western countries are idiopathic, prior cardiac surgery, and mediastinal radiation [1]. Although uncommon, blunt trauma should be considered as an initiating cause of CP and can have a delayed presentation [2]. MMA is a contact heavy sport causing blunt trauma, based upon standing combat, grappling and ground fighting, and striking opponents by incorporating various martial arts techniques from around the world. The cardiac risks facing MMA athletes is largely unknown, since studies tend to be focused on musculoskeletal injury or head trauma [3]. Therefore, objectives of the case are as follows: to assist readers in recognizing MMA as a risk factor for CP; to diagnose CP that is potentially secondary to recurring trauma leading to hemorrhagic effusions; to recognize that in the case of repeated blunt trauma, delayed presentation of CP may be seen; to identify liver abnormalities secondary to right heart failure as a unique presentation of CP in addition to dyspnea on exertion and peripheral edema; and to select appropriate testing for CP in the setting of elevated jugular venous pressure and liver abnormalities.

## Case presentation

A 47-year-old Caucasian male former MMA fighter from Nevada presented to liver clinic for elevated liver injury tests (LIT) and a 35-pound weight loss associated with nausea, vomiting, and diarrhea. He endorsed symptoms of lower extremity edema, dyspnea on exertion, and fatigue which had worsened over 6 months and was treated with diuretics. Physical exam revealed jugular venous pressure (JVP) of 15 cm with a steep *y* descent, without Kussmaul’s sign. He had hepatomegaly with a firm liver edge 4 cm below the right costal margin. He had normal S1 and S2 without additional sounds. He had no evidence of pleural effusion or ascites but did have trace peripheral edema.

**Fig. 1:**
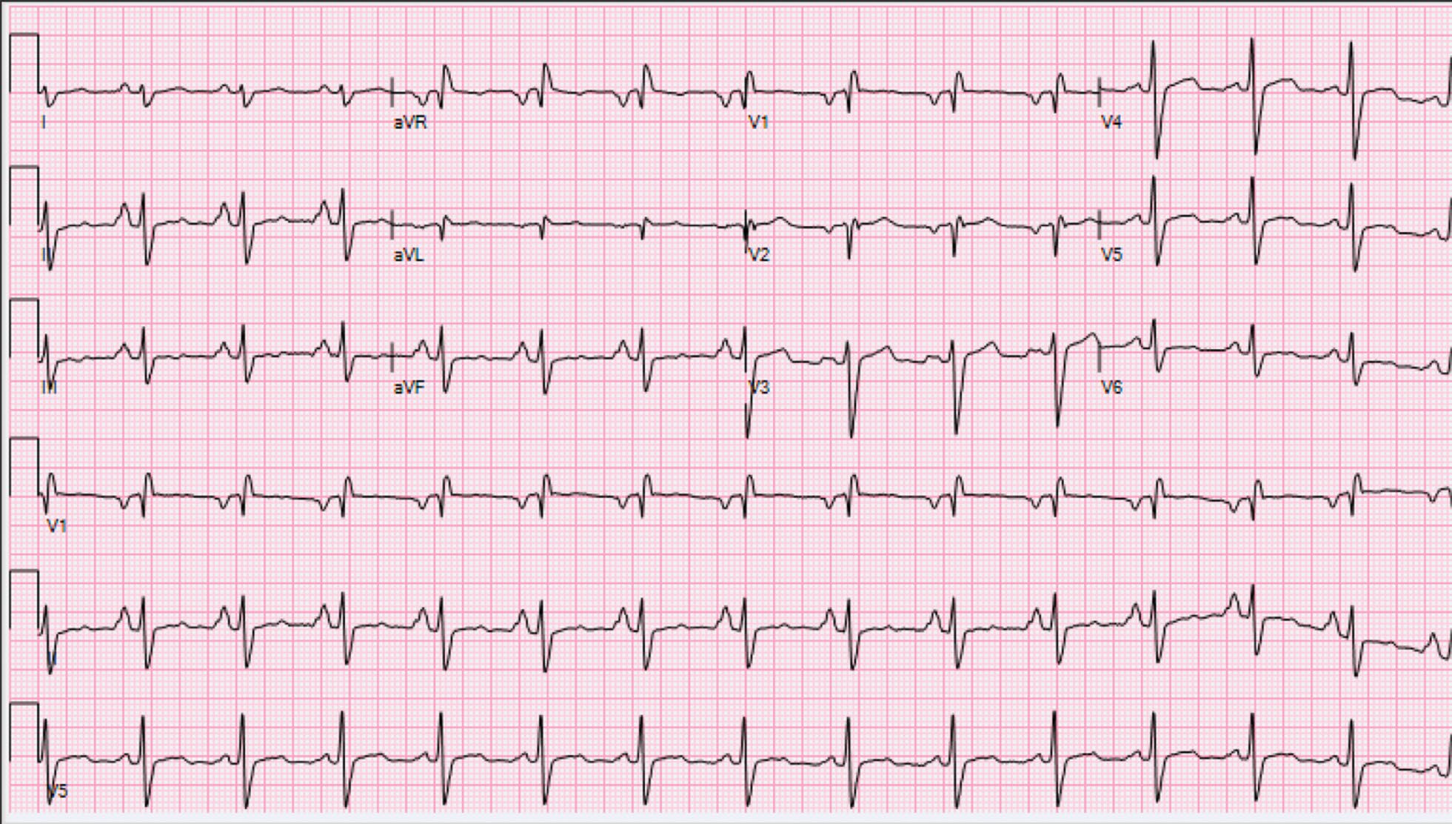
Electrocardiogram demonstrating biatrial enlargement and right bundle branch block

The patient has a past medical history of concussion, migraine headache, hypertension, chronic pain related to musculoskeletal injuries and fractures secondary to MMA competition, and right bundle branch block (Fig. 1). Past surgical history is notable for fracture surgeries, diagnostic right knee scope, right shoulder surgery, and foot surgery. He reported a 20-year history of performance enhancing drugs (PEDs), including testosterone use that ceased 8 months prior to presentation. Notably, the patient had an 8-year history of being a professional MMA fighter and a 13-year history of MMA fighting with recurrent trauma to the chest wall.

**Fig. 2:**
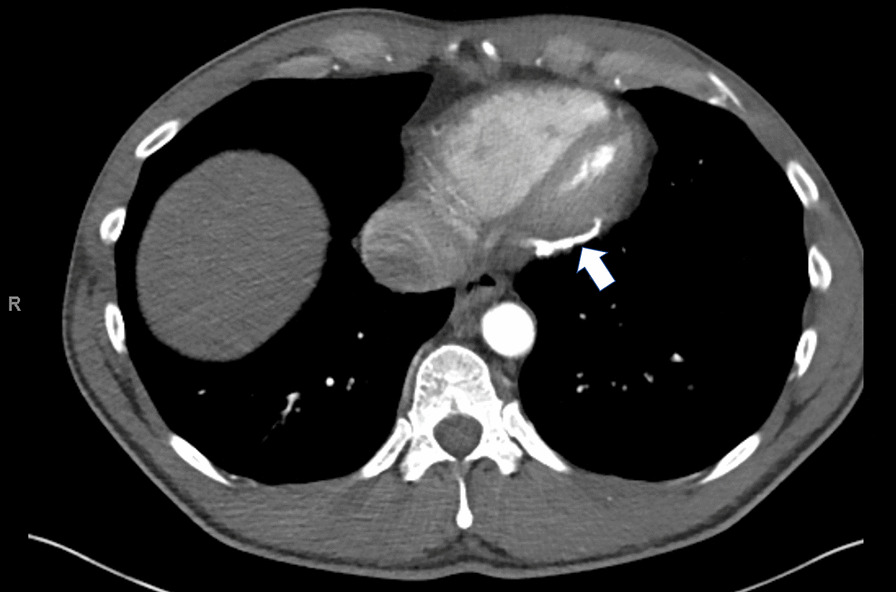
Computed tomography demonstrates pericardial calcification (white arrow)

**Fig. 3:**
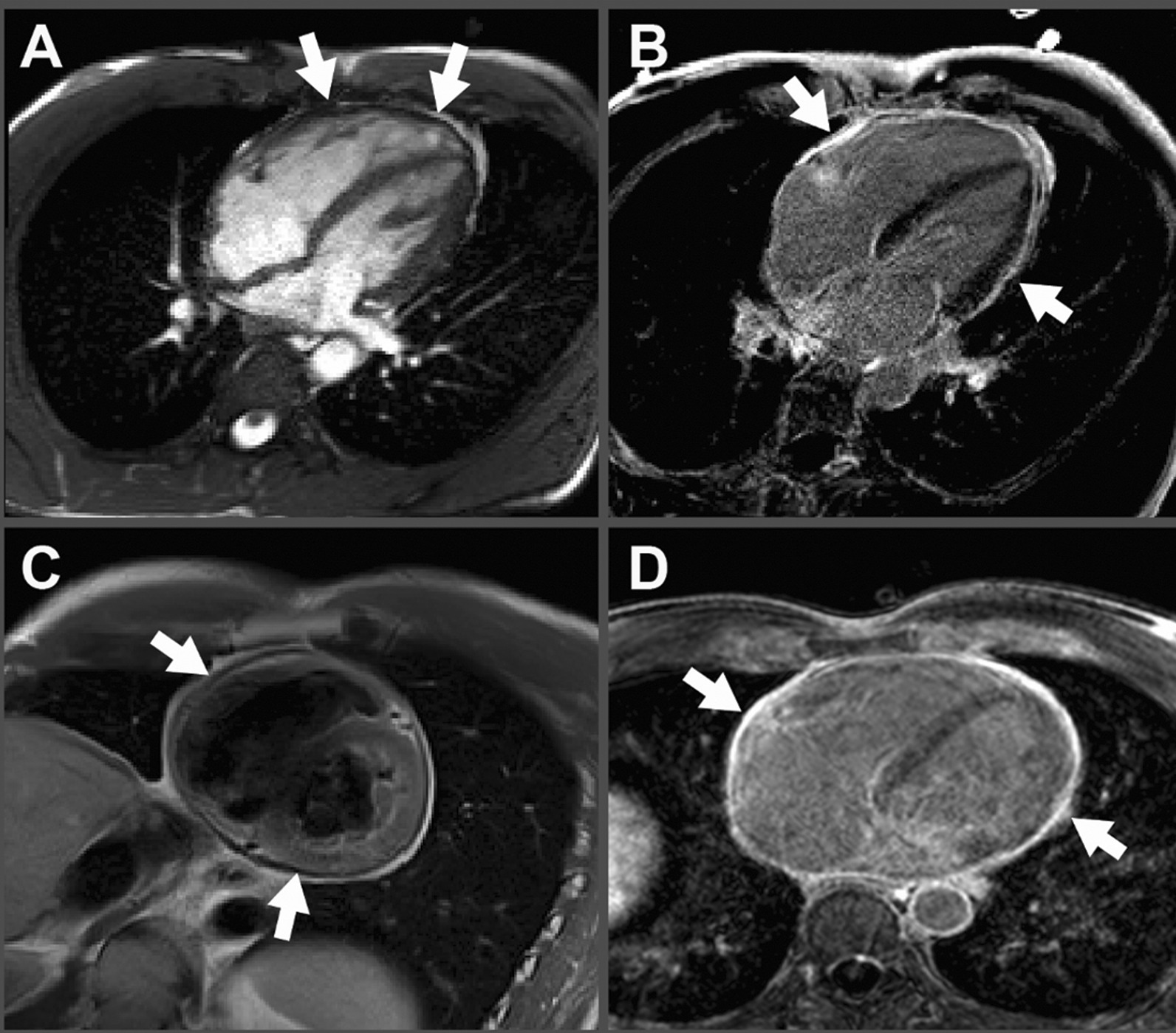
Cardiac magnetic resonance imaging. **A** Steady state free precession (“cine”) imaging demonstrates circumferential thickening of the pericardium (indicated with white arrows) and significant signal void consistent with calcification **B** Phase sensitive inversion recovery imaging approximately 10 min after administration of gadobenate dimeglumine demonstrates marked circumferential late gadolinium enhancement (indicated with white arrows) suggesting presence of inflamed and/or scar tissue **C** Double inversion recovery (“black blood”) imaging demonstrates marked circumferential thickening of the pericardium (indicated with white arrows) **D** Whole heart high resolution 3D inversion recovery imaging demonstrates pronounced late gadolinium enhancement of the entire pericardium (indicated with white arrows). No significant late enhancement of the myocardium was seen, suggesting lack of myocardial involvement or that any associated inflammatory process involving the myocardium has resolved without myocardial scar formation

**Fig. 4:**
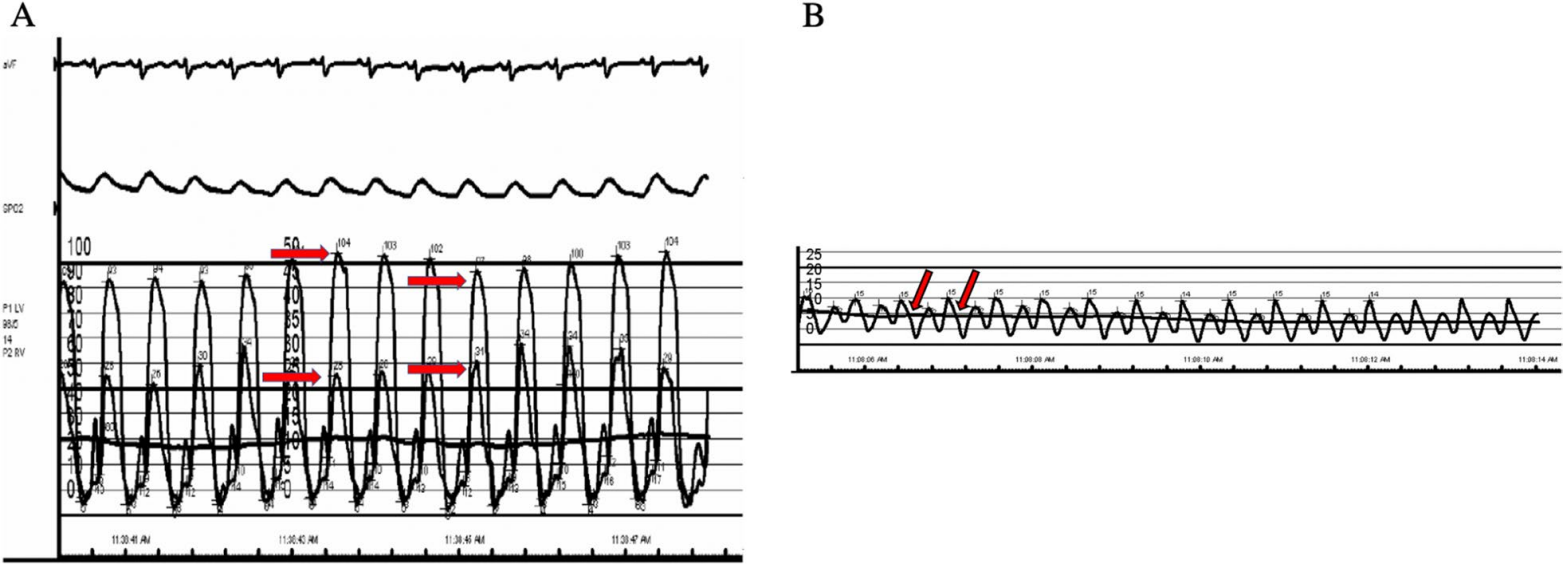
**A** Simultaneous left ventricular and right ventricular pressure recording shows discordance in the ventricular peak systolic pressures during respiration (red arrows). **B** Right atrial pressure wave form, demonstrating the steep *y* descent (red arrows)

Differential diagnosis included pulmonary hypertension, chronic pulmonary emboli, heart failure, cirrhosis, and constrictive pericarditis. As patient initially presented to liver clinic, preliminary work-up showed hepatic congestion with ALT and AST of 1155 U/L and 219 U/L respectively. Abdominal computerized tomography demonstrated hepatomegaly with steatosis, ascites, a dilated inferior vena cava, and a calcified pericardium (Fig. 2). During liver biopsy, the patient had documented right atrial pressure (RAP) of 12 mmHg, free hepatic vein pressure of 12 mmHg, wedged sinusoidal pressure of 13 mmHg, and hepatic vein portal vein gradient of 1 mmHg, diagnostic of post hepatic portal hypertension secondary to right heart failure. These findings warranted referral to cardiology, where echocardiogram revealed abnormal septal motion suggestive of ventricular interdependence (Additional file 1: Video S1, Additional file 2: Video S2, Additional file 3: Video S3). Cardiac magnetic resonance imaging (MRI) revealed marked thickening of the pericardium, MRI signal void consistent with pericardial calcification, and profound late gadolinium enhancement on both standard and high-resolution inversion recovery images. There was no significant myocardial late gadolinium enhancement, suggesting that any potential inflammatory involvement of the myocardium has resolved without myocardial scar formation (Fig. 3). Right and left heart catheterization revealed normal coronary arteries and normal pulmonary artery pressures (Table 1) with right ventricle-left ventricle (RV-LV) discordance, consistent with constrictive physiology (Fig. 4).

**Table 1:** Right heart catheterization hemodynamic data

Right atrial pressure	15 mmHg
Right ventricular pressure	38/12 mmHg
Pulmonary artery pressure (mean)	36/12 (20) mmHg
Pulmonary capillary wedge pressure	12 mmHg
Systolic blood pressure	101/68 mmHg
Cardiac output	6.23 L/min
Cardiac index	2.89 L/min/m^2^
Left ventricular end-diastolic pressure	13 mmHg
Pulmonary vascular resistance	1.28 Wood Units
Systemic vascular resistance	1181.38 dynes * sec/cm^5^

**Fig. 5:**
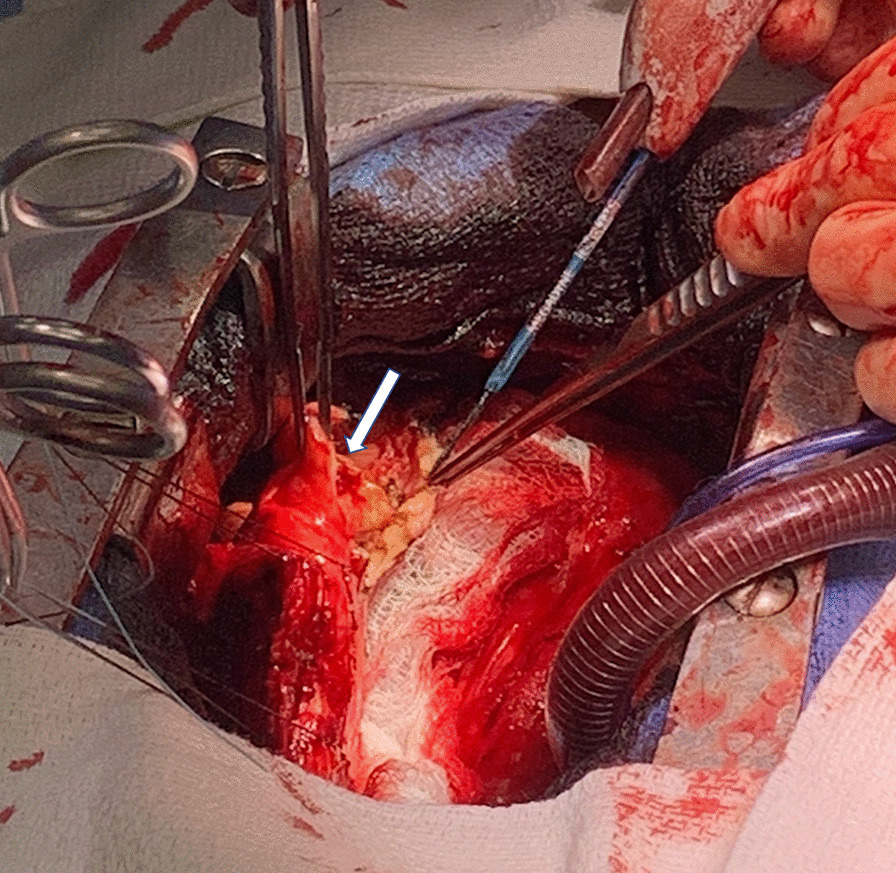
Intraoperative findings of markedly thickened pericardium (white arrow)

**Fig. 6:**
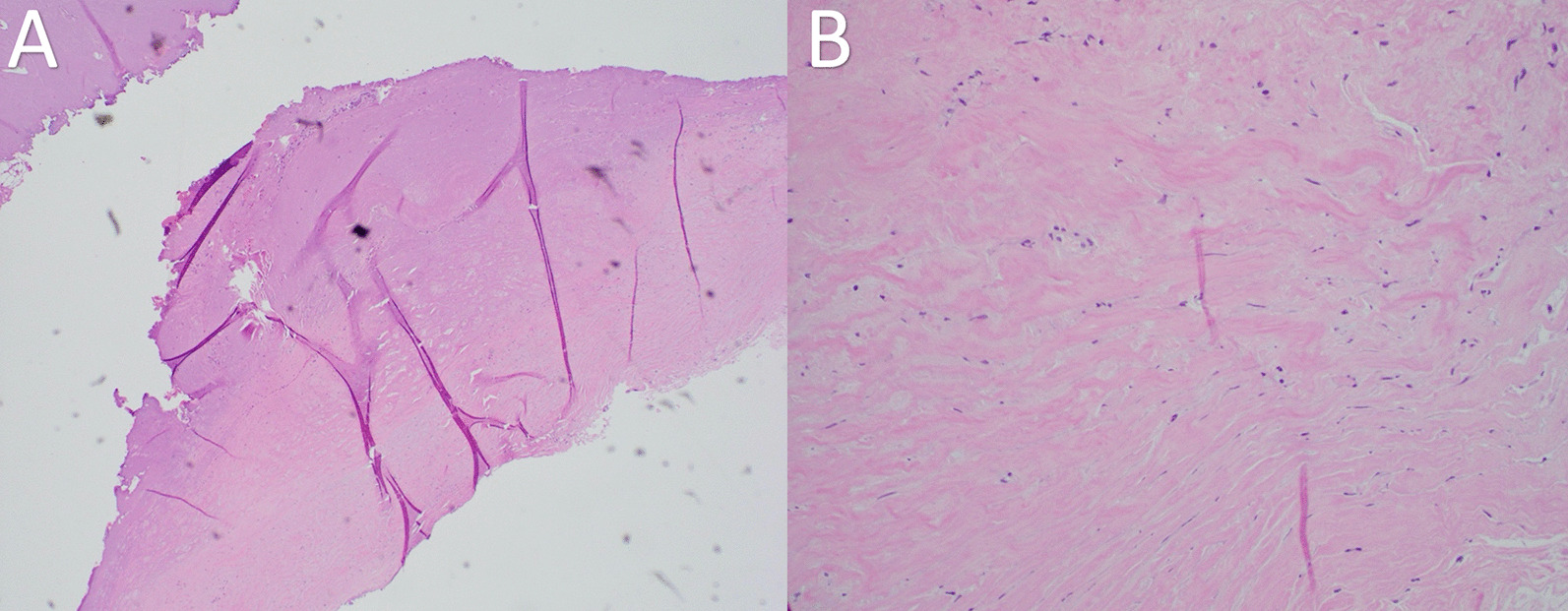
Pericardial histology at 20× demonstrating fibrosis and mild chronic inflammation consistent with chronic pericarditis. **A** 4× demonstrating fibrosis and chronic inflammation **B** 20× demonstrating fibrosis and chronic inflammation

Pericardiectomy was performed via sternotomy with cardiopulmonary bypass. During the procedure, calcification and thickening of the pericardium was evident (Fig. 5). The pericardium was removed from phrenic-to-phrenic nerve anteriorly and was removed in the oblique sinus and posterior to the phrenic nerve on the left. Histology confirmed pericardial tissue with fibrosis and inflammation consistent with chronic pericarditis (Fig. 6). These changes can be seen in chronic pericarditis of various etiologies; however, there was no evidence of acute or granulomatous inflammation, and the dense fibrosis without chronic inflammation implicated repeated trauma as a potential etiology, where in the setting of lack of other risk factors, appears to be the likely cause. Fungal and bacterial cultures were negative.

After pericardiectomy, the patient’s hospital course was uncomplicated. At his 7-week post-operative clinic visit, the patient reported he had returned to jogging and no longer had any of his previous symptoms of lower extremity edema, dyspnea on exertion, fatigue, diarrhea secondary to small bowel edema, or hepatic congestion. In the absence of poor predictors, a good prognosis is anticipated, and patient is now NYHA functional class I.

## Discussion and conclusions

The most common etiologies of CP in Western countries are idiopathic, prior cardiac surgery, and mediastinal radiation [1]. Infectious or inflammatory causes do not appear to be the etiology in this case. The patient did not describe any syndrome of acute pericarditis and did not live in a region where, for example, tuberculosis is more prevalent. His hepatitis screening was negative. He also did not have clinical or serological evidence of autoimmune disease, despite a thorough work-up, no history of cardiac surgery, radiation, or relevant infections, but does notably have 20 years of repeated chest trauma due to MMA fighting, thereby implicating repeated chest trauma as the most likely etiology for his CP. Admittedly, pericardial thickening and calcification often represents a burned-out stage in pericarditis regardless of etiology, by the time a patient undergoes pericardiectomy, there may be little evidence of active inflammation.

MMA is certainly a cause of recurrent blunt trauma, as it is a contact heavy sport based upon standing combat, grappling and ground fighting, and striking opponents by incorporating various martial arts techniques. Little is known regarding cardiac risks for MMA fighters, with the majority of studies has been focused on musculoskeletal or head injuries [3]. In this case, the patient stated that he was kicked, kneed, and punched in the ribs approximately 50 times in a 2-h practice, occurring 3 to 4 times a week for several years. Although uncommon, blunt trauma should be considered as a potential initiating cause of CP and can have a delayed presentation [2, 4]. This rare phenomenon has been documented sporadically in the literature, most notably in the case of a boxer with recurrent trauma to the chest wall [5]. This case expands our understanding of the physical consequences of MMA fighting and raises additional questions about the clinical sequelae of PEDs in sports [6, 7].

Right and left heart catheterization remain the gold standard in the diagnosis of CP and is primarily characterized by the near equalization of end-diastolic pressures in all chambers and the pulmonary artery [8] (Table 1). In addition to RV-LV discordance on catheterization, right atrial tracing may demonstrate a prominent *y* descent (Fig. 4B). Transthoracic echocardiogram (TTE) has been shown to be diagnostic of CP in 70% of cases. Both cardiac MRI and TTE are capable of distinguishing CP from other forms of heart failure, including restrictive cardiomyopathy [9–11], as in this case presented here.

The 5-year survival after pericardiectomy ranges from 65 to 90% depending on etiology. Predictors of poor outcome are prior radiation, renal insufficiency, poor ventricular function, higher pulmonary artery pressures, NYHA class IV, low serum sodium, ascites, and hyperbilirubinemia, all of which were absent in this patient [12].

CP can be a sequela of recurrent pericarditis or hemorrhagic effusions and may have a delayed presentation. Here, we present a case of CP most likely caused by recurrent trauma to the chest wall secondary to MMA fighting. In cases of recurrent chest wall trauma, CP may be managed with pericardiectomy with apparent good outcome. Further studies are warranted to analyze the occurrence of CP in MMA to better define the risk in such adults. In the case of individuals who have experienced recurrent or significant chest wall trauma, there must be a higher index of suspicion when considering CP on the differential diagnosis.

## Supplementary Information

**Additional file 1: Video S1.** Transthoracic echocardiogram (TTE) demonstrates abnormal septal motion indicative of ventricular interdependence.

**Additional file 2: Video S2.** TTE Parasternal Long axis demonstrates septal bounce.

**Additional file 3: Video S3.** TTE Parasternal Short axis demonstrates septal bounce.

## Data Availability

The datasets during and/or analyzed during the current study are included within the manuscript and the supporting files. Additional data will be made available from the corresponding author on reasonable request.

## Abbreviations

ALT: Aspartate transaminase
AST: Alanine transaminase
CP: Constrictive pericarditis
CT: Computed tomography
PED: Performance enhancing drug
ECG: Electrocardiogram
LV: Left ventricle
RV: Right ventricle
LIT: Liver injury tests
MMA: Mixed martial arts
MRI: Magnetic resonance imaging
NYHA: New York Heart Association
RAP: Right atrial pressure
TTE: Transthoracic echocardiogram
